# Special Issue: Epstein–Barr–Virus–Associated Cancers

**DOI:** 10.3390/microorganisms9020241

**Published:** 2021-01-25

**Authors:** Asuka Nanbo

**Affiliations:** Research Center for the Control and Prevention of Infectious Diseases, Nagasaki University, 1-12-4, Sakamoto, Nagasaki 852-8523, Japan; nanboa@nagasaki-u.ac.jp

Epstein–Barr virus (EBV), a ubiquitous human gamma herpesvirus, infects a majority of the population worldwide (~95%). Although EBV infections are persistent and lifelong, they are largely asymptomatic. However, the virus can cause various lymphoid and epithelial malignancies, such as Burkitt’s lymphoma, Hodgkin lymphoma (HL), gastric carcinoma (GC), and nasopharyngeal carcinoma (NPC). Since its discovery as the first human oncovirus in 1964, accumulating evidence indicates that a variety of viral factors, including oncoproteins and non-coding RNAs, contribute to the development of EBV-associated cancers by regulating diverse cellular machineries [1]. Despite extensive research, the underlying mechanisms by which EBV causes cancers in only a restricted population remains unclear.

This Special Issue assembles eleven papers, comprising seven research articles, two reviews, and two brief reports, which collectively provide novel information on the mechanisms of oncogenesis [2,3,4,5,6] and viral replication [7,8,9,10], as well as applications for diagnosis [11,12].

Using a variety of methods and approaches, several studies have provided a novel notion in understanding how EBV contributes to the development of various tumors, including GC [2,3], HL [4], NPC [5], and oral squamous cell carcinoma [6], on a molecular basis. A distinct feature of EBV-associated GC is the significant infiltration of immune cells in the epithelium, which appears to establish a unique tumor microenvironment. A study demonstrated that exosomes induce the suppression of maturation of infiltrated dendritic cells in EBV-associated GC [2]. Bioinformatic approaches revealed that an EBV-encoded microRNA, miR-BART17-5p, directly downregulates Kruppel-like factor 2 in EBV-associated GC cells, which subsequently confers an oncogenic phenotype [3]. Other studies identified host factors such as Notch1 and Pax5 or Semaphorin 3A, which exhibited correlated expression with viral infection in EBV-associated HL [4] or NPC [5], respectively, indicating that they could be considered as a candidate diagnostic/prognostic marker and a therapeutic target in these tumors. EBV establishes latent infection in squamous cell carcinoma cell lines, suggesting that it can be used as an in vitro model to investigate the role of EBV in the development of uncharacterized oral squamous cell carcinoma [6].

Some studies have broadened our knowledge of the maturation of EBV particles. One study characterized the role of EBV-encoded BBRF2 in the viral lytic cycle by using a knockout virus. Although the viral gene expression, genome propagation, and progeny production were not affected by deletion of the BBRF2 gene, infectivity of the progeny viruses was reduced. Further analysis demonstrated that BBRF2 protects its counter partner, BSRF1, from proteasome/ubiquitin-mediated degradation [7]. Another study using microscopic approaches provided further insights into the EBV lifecycle by demonstrating that EBV exploits the host secretion pathway for the release of progeny virions into the extracellular milieu [8].

Furthermore, two studies introduced novel approaches for the diagnosis of EBV-associated GC. The contribution of *Helicobacter pylori*-induced persistent gastric mucosal ulceration damage in EBV-associated GC was evaluated using the rapid urease test to measure viral copy numbers in gastric biopsy samples with atrophic gastritis. A significant correlation was found between high viral copy numbers and severe grades of atrophic gastritis [11]. A droplet digital PCR-based screening method was established to assess the EBV infection status in the GC specimens, which was validated through complete correlation with a conventional EBER1 in situ hybridization assay [12].

Finally, the two reviews highlight the contribution of the viral lytic cycle in EBV-associated diseases. One provides recent knowledge about EBV-induced autoimmune diseases, which is likely mediated by the upregulation of antibody secretion from latently infected B cells upon EBV reactivation [9]. The second is a systematic review discussing the recent advances in understanding the molecular mechanism by which lytic cycle of EBV contributes to tumor formation and progression [10].

Collectively, this Special Issue presents significant progress in the understanding of how EBV induces oncogenesis in various ways; nevertheless, the fundamental priority is yet to be addressed. Further studies are necessary to enable the development of therapeutics to control EBV-associated diseases.
